# (3-Chloro­phen­yl){2-eth­oxy-5-[(*Z*)-hydroxy(phen­yl)methyl­idene]cyclo­penta-1,3-dien-1-yl}methanone

**DOI:** 10.1107/S1600536811055279

**Published:** 2012-01-11

**Authors:** Mihaela-Liliana Tinţaş, Richard A. Varga, Ion Grosu, Elena Bogdan

**Affiliations:** a Organic Chemistry Department, Faculty of Chemistry and Chemical Engineering, Babeş-Bolyai University, Arany Janos 11, 400028, Cluj Napoca, Romania; b Inorganic Chemistry Department, Faculty of Chemistry and Chemical Engineering, Babeş-Bolyai University, Arany Janos 11, 400028, Cluj Napoca, Romania

## Abstract

The title compound, C_21_H_17_ClO_3_, which crystallizes as one of two possible oxo/hy­droxy-fulvene prototropic tautomers, possesses a strong intra­molecular O—H⋯O hydrogen bond that closes a seven-membered ring. The dihedral angles between the central five-membered ring and two pendant rings are 55.05 (9) and 44.51 (10)°. The crystal packing is characterized by weak inter­molecular C—H⋯O inter­actions between an H atom of the oxymethyl­ene unit and the carbonyl group of an adjacent mol­ecule, resulting in formation of chains of mol­ecules along the *a* axis.

## Related literature

For the structures of related 2-acyl-6-hydoxyfulvene derivatives, see: Ferguson *et al.* (1975 ▶); Dong *et al.* (2004 ▶, 2006 ▶). For more information on the synthesis of 2-acyl-6-hydoxyfulvene derivatives, see: Dong *et al.* (2004 ▶, 2006 ▶). For preparation details, see: Christl *et al.* (1998 ▶). For compounds obtained from 2-acyl-6-hydoxyfulvenes, see: Dong *et al.* (2004 ▶); Li *et al.* (2008 ▶); Snyder *et al.* (2005 ▶). For complexes based on 2-acyl-6-hydoxyfulvenes, see: Dong *et al.* (2004 ▶, 2006 ▶); Wang *et al.* (2005 ▶). For their various applications, see: Hong *et al.* (2005 ▶); Kondo *et al.* (1992 ▶); Vicente *et al.* (1995 ▶).
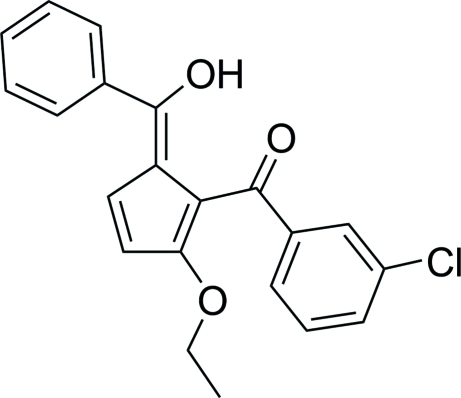


## Experimental

### 

#### Crystal data

C_21_H_17_ClO_3_
*M* *_r_* = 352.80
Monoclinic, 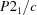

*a* = 8.1369 (16) Å
*b* = 27.737 (6) Å
*c* = 7.6709 (15) Å
β = 98.51 (3)°
*V* = 1712.2 (6) Å^3^
*Z* = 4
Mo *K*α radiation
μ = 0.24 mm^−1^
*T* = 297 K
0.30 × 0.29 × 0.26 mm

#### Data collection

Bruker SMART APEX CCD area-detector diffractometer
Absorption correction: multi-scan (*SADABS*; Bruker, 2000 ▶) *T*_min_ = 0.932, *T*_max_ = 0.940
15678 measured reflections
3002 independent reflections
2683 reflections with *I* > 2σ(*I*)
*R*_int_ = 0.040

#### Refinement

*R*[*F* ^2^ > 2σ(*F* ^2^)] = 0.061
*wR*(*F* ^2^) = 0.125
*S* = 1.17
3002 reflections
231 parameters
H atoms treated by a mixture of independent and constrained refinement
Δρ_max_ = 0.22 e Å^−3^
Δρ_min_ = −0.29 e Å^−3^

### 

Data collection: *SMART* (Bruker, 2000 ▶); cell refinement: *SAINT-Plus* (Bruker, 2000 ▶); data reduction: *SAINT-Plus*; program(s) used to solve structure: *SHELXS97* (Sheldrick, 2008 ▶); program(s) used to refine structure: *SHELXL97* (Sheldrick, 2008 ▶); molecular graphics: *DIAMOND* (Brandenburg & Putz, 2006 ▶); software used to prepare material for publication: *publCIF* (Westrip, 2010 ▶).

## Supplementary Material

Crystal structure: contains datablock(s) I, global. DOI: 10.1107/S1600536811055279/ld2041sup1.cif

Structure factors: contains datablock(s) I. DOI: 10.1107/S1600536811055279/ld2041Isup2.hkl

Supplementary material file. DOI: 10.1107/S1600536811055279/ld2041Isup3.cml

Additional supplementary materials:  crystallographic information; 3D view; checkCIF report

## Figures and Tables

**Table 1:** Hydrogen-bond geometry (Å, °)

*D*—H⋯*A*	*D*—H	H⋯*A*	*D*⋯*A*	*D*—H⋯*A*
O1—H1⋯O2	1.07 (5)	1.38 (5)	2.435 (3)	168 (5)
C20—H20*B*⋯O2^i^	0.97	2.51	3.246 (3)	133

Symmetry code: (i) 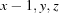.

**Fig. 1.**
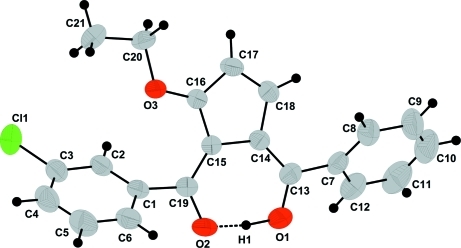
The structure of the title compound, showing the atom-labelling scheme and the intramolecular O1—H1···O2 hydrogen bond. Displacement ellipsoids are shown at the 50% probability level.

**Fig. 2.**
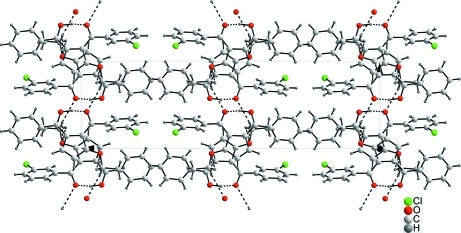
Packing diagram for the crystal of the title compound viewed along c axis.

## supplementary crystallographic information

### Comment

Widely used as synthetic precursors, fulvenes are key intermediates in the total synthesis of several natural products (Hong *et al.* 2005, Vicente *et al.* 1995) and are well known as one of the important organic ligands used in construction of organometalic complexes (Li *et al.* 2008, Dong *et al.* 2006, Snyder *et al.* 2005, Wang *et al.* 2005, Dong *et al.* 2004). Fulvenes also raised interest due to their potential as non-linear optic materials (Kondo *et al.* 1992).

The title compound was obtained as a by-product in the oxidation reaction of dihydro-α-pyrone 1-(3-chlorophenyl)-4-phenyl-4a,5-dihydrocyclopenta[*c*]pyran-3(4*H*)-one with DDQ (2,3-dichloro-5,6-dicyano-1,4-benzoquinone) in chloroform (Christl *et al.* 1998). We observed that during the dehydrogenation process in order to obtain the corresponding α-pyrone, a competitive reaction underwent with formation of the fulvene derivative (Fig. 1) only when chloroform containing 0.5–1% ethanol as stabilizer is used as solvent. By performing the oxidation reaction in ethanol free of chloroform, no fulvene structure of the title compound could be identified.

In the molecule of the title compound (Fig. 1), the C—C bond lenghts of the five-membered ring as well as C13—C14 and C15—C19 range from 1.366 (3) to 1.481 (7) Å, corresponding to a delocalized system extended from C═O to the enol OH group (Ferguson *et al.* 1975). The hydroxy-fulvene tautomer is favored by the strong intramolecular H-bonding of the enol hydrogen atom H1 and the carbonyl oxygen atom O2, [H1···O2 = 1.38 (5) Å, O1—H1···O2 = 168 (5)°]. Thus a H-bonded seven-membered ring almost coplanar with the five-membered one is formed. The two aryl units attached at C13 and C19 are almost orthogonal disposed, the dihedral angles between the phenyl and *m*-chlorophenylene with respect to the seven-membered ring plane being 44.4 (9) and 54.4 (0)° respectively.

The crystal packing (Fig. 2) is stabilized by weak intermolecular C—H···O hydrogen bonds, therefore chains of parallel molecules having the same orientation are formed [H20B···O2^i^ = 2.51 (2) Å, C20—H20B···O2^i^ = 133 (2)°; symmetry code: (i) -1 + *x*, *y*, *z*].

### Experimental

A solution of (4*S*,4a*R*)-1-(3-chlorophenyl)-4-phenyl-4a,5-dihydrocyclopenta[*c*]pyran-3(4*H*)-one (0.5 g, 1.55 mmol s, 1 equiv.), 2,3-dichloro-5,6-dicyano-1,4-benzoquinone in 10 ml anhydrous chloroform (with 0.5–1% ethanol as stabilizer) was refluxed under argon for 1.5 h. The reaction mixture was cooled to 0 °C and the brown precipitate was filtered-off. The filtrate was washed with water and brine, dried with MgSO_4_ and the solvent removed *in vacuo* at 30 °C. The crude product was purified by column chromatography on silica gel (petroleum ether/diethyl ether = 2:1) to afford the major product 1-(3-chlorophenyl)-4-phenylcyclopenta[*c*]pyran-3(5*H*)-one (0.094 g, 19%) as a yellow solid, and (*Z*)-(3-chlorophenyl)(2-ethoxy-5-(hydroxy(phenyl)methylene)cyclopenta-1,3-dien-1-yl)methanone (0.042 g, 8%) as a light brown solid. Crystals suitable for the diffraction experiment were obtained by slow evaporation from solution (pethroleum ether / diethyl ether = 1: 2) of the title compound.

### Refinement

All C-bound H atoms were placed in calculated positions (C—H = 0.93–0.97 Å) and treated using a riding model with *U*_iso_= 1.5*U*_eq_(C) for methyl H atoms. The methyl group was allowed to rotate, but not to tip, to best fit the electron density. Although O1 and O2 are chemically almost equivalent as protonation sites (the only difference is the position of a remote OEt and Cl substituents), the hydrogen was objectively localized and refined at O1. Its high thermal parameter is an attribute of a strong intramolecular H bond and our attempt to refine a model with the hydrogen disordered between the two alternative positions was unsuccessful (*i.e*. this is a single-well potential surface for the proton - at least at room temperature).

### Crystal data

**Table:**

C_21_H_17_ClO_3_	*F*(000) = 736
*M**_r_* = 352.80	*D*_x_ = 1.369 Mg m^−^^3^
Monoclinic, *P*2_1_/*c*	Mo *K*α radiation, λ = 0.71073 Å
Hall symbol: -P 2ybc	Cell parameters from 3314 reflections
*a* = 8.1369 (16) Å	θ = 2.5–23.2°
*b* = 27.737 (6) Å	µ = 0.24 mm^−^^1^
*c* = 7.6709 (15) Å	*T* = 297 K
β = 98.51 (3)°	Block, yellow
*V* = 1712.2 (6) Å^3^	0.30 × 0.29 × 0.26 mm
*Z* = 4	

### Data collection

**Table:**

Bruker SMART APEX CCD area-detector diffractometer	3002 independent reflections
Radiation source: fine-focus sealed tube	2683 reflections with *I* > 2σ(*I*)
graphite	*R*_int_ = 0.040
phi and ω scans	θ_max_ = 25.0°, θ_min_ = 2.5°
Absorption correction: multi-scan (*SADABS*; Bruker, 2000)	*h* = −9→9
*T*_min_ = 0.932, *T*_max_ = 0.940	*k* = −32→32
15678 measured reflections	*l* = −9→9

### Refinement

**Table:**

Refinement on *F*^2^	Primary atom site location: structure-invariant direct methods
Least-squares matrix: full	Secondary atom site location: difference Fourier map
*R*[*F*^2^ > 2σ(*F*^2^)] = 0.061	Hydrogen site location: inferred from neighbouring sites
*wR*(*F*^2^) = 0.125	H atoms treated by a mixture of independent and constrained refinement
*S* = 1.17	*w* = 1/[σ^2^(*F*_o_^2^) + (0.0346*P*)^2^ + 1.0364*P*] where *P* = (*F*_o_^2^ + 2*F*_c_^2^)/3
3002 reflections	(Δ/σ)_max_ < 0.001
231 parameters	Δρ_max_ = 0.22 e Å^−^^3^
0 restraints	Δρ_min_ = −0.29 e Å^−^^3^

### Special details

**Table:**

Geometry. All e.s.d.'s (except the e.s.d. in the dihedral angle between two l.s. planes) are estimated using the full covariance matrix. The cell e.s.d.'s are taken into account individually in the estimation of e.s.d.'s in distances, angles and torsion angles; correlations between e.s.d.'s in cell parameters are only used when they are defined by crystal symmetry. An approximate (isotropic) treatment of cell e.s.d.'s is used for estimating e.s.d.'s involving l.s. planes.
Refinement. Refinement of *F*^2^ against ALL reflections. The weighted *R*-factor *wR* and goodness of fit *S* are based on *F*^2^, conventional *R*-factors *R* are based on *F*, with *F* set to zero for negative *F*^2^. The threshold expression of *F*^2^ > 2σ(*F*^2^) is used only for calculating *R*-factors(gt) *etc*. and is not relevant to the choice of reflections for refinement. *R*-factors based on *F*^2^ are statistically about twice as large as those based on *F*, and *R*- factors based on ALL data will be even larger.

### Fractional atomic coordinates and isotropic or equivalent isotropic displacement parameters (Å^2^)

**Table:**

	*x*	*y*	*z*	*U*_iso_*/*U*_eq_	
Cl1	0.18516 (11)	0.68353 (3)	0.59613 (12)	0.0710 (3)	
O1	0.4203 (2)	0.43102 (8)	0.1762 (3)	0.0672 (6)	
O2	0.4234 (2)	0.51880 (8)	0.1798 (4)	0.0765 (7)	
C15	0.1504 (3)	0.50390 (9)	0.2264 (3)	0.0368 (6)	
C19	0.2853 (3)	0.53450 (10)	0.2149 (4)	0.0466 (7)	
C14	0.1450 (3)	0.45072 (9)	0.2082 (3)	0.0374 (6)	
C16	−0.0122 (3)	0.51749 (9)	0.2502 (3)	0.0352 (5)	
C17	−0.1124 (3)	0.47648 (9)	0.2510 (3)	0.0424 (6)	
H17	−0.2239	0.4761	0.2651	0.051*	
C1	0.2831 (3)	0.58783 (9)	0.2398 (4)	0.0428 (6)	
C2	0.2385 (3)	0.60855 (9)	0.3890 (4)	0.0452 (6)	
H2	0.2033	0.5894	0.4757	0.054*	
C18	−0.0174 (3)	0.43706 (9)	0.2275 (3)	0.0417 (6)	
H18	−0.0553	0.4054	0.2246	0.050*	
C3	0.2463 (3)	0.65780 (10)	0.4090 (4)	0.0486 (7)	
C13	0.2684 (3)	0.41810 (10)	0.1820 (3)	0.0442 (6)	
C7	0.2423 (3)	0.36553 (9)	0.1635 (3)	0.0435 (6)	
C6	0.3382 (3)	0.61725 (11)	0.1155 (4)	0.0534 (7)	
H6	0.3711	0.6037	0.0153	0.064*	
C4	0.2980 (4)	0.68732 (11)	0.2852 (5)	0.0608 (8)	
H4	0.3012	0.7206	0.3003	0.073*	
C8	0.1071 (4)	0.34512 (10)	0.0608 (4)	0.0539 (7)	
H8	0.0261	0.3649	−0.0002	0.065*	
C5	0.3449 (4)	0.66657 (11)	0.1384 (5)	0.0634 (9)	
H5	0.3818	0.6859	0.0531	0.076*	
C12	0.3628 (4)	0.33490 (11)	0.2487 (4)	0.0604 (8)	
H12	0.4573	0.3477	0.3157	0.073*	
C10	0.2075 (5)	0.26619 (12)	0.1346 (5)	0.0813 (11)	
H10	0.1952	0.2329	0.1260	0.098*	
C9	0.0901 (5)	0.29571 (11)	0.0471 (5)	0.0705 (9)	
H9	−0.0024	0.2826	−0.0225	0.085*	
C11	0.3434 (5)	0.28564 (13)	0.2348 (5)	0.0786 (11)	
H11	0.4240	0.2655	0.2946	0.094*	
O3	−0.0580 (2)	0.56340 (6)	0.2675 (2)	0.0435 (4)	
C20	−0.2232 (3)	0.57156 (10)	0.3075 (4)	0.0461 (7)	
H20A	−0.2425	0.5518	0.4070	0.055*	
H20B	−0.3049	0.5631	0.2070	0.055*	
C21	−0.2378 (4)	0.62365 (11)	0.3506 (4)	0.0577 (8)	
H21A	−0.1538	0.6320	0.4475	0.087*	
H21B	−0.3456	0.6298	0.3825	0.087*	
H21C	−0.2234	0.6428	0.2497	0.087*	
H1	0.436 (5)	0.4691 (17)	0.188 (6)	0.127 (16)*	

### Atomic displacement parameters (Å^2^)

**Table:**

	*U*^11^	*U*^22^	*U*^33^	*U*^12^	*U*^13^	*U*^23^
Cl1	0.0812 (6)	0.0519 (5)	0.0806 (6)	0.0001 (4)	0.0148 (4)	−0.0210 (4)
O1	0.0454 (12)	0.0493 (13)	0.1120 (19)	0.0028 (10)	0.0289 (12)	−0.0124 (12)
O2	0.0396 (11)	0.0527 (13)	0.145 (2)	−0.0082 (10)	0.0392 (13)	−0.0193 (14)
C15	0.0341 (13)	0.0366 (14)	0.0405 (14)	−0.0013 (10)	0.0079 (11)	−0.0027 (11)
C19	0.0372 (15)	0.0462 (16)	0.0584 (17)	−0.0032 (12)	0.0133 (13)	−0.0069 (13)
C14	0.0377 (14)	0.0361 (14)	0.0391 (14)	−0.0024 (11)	0.0080 (11)	0.0017 (11)
C16	0.0337 (12)	0.0357 (14)	0.0363 (13)	−0.0013 (11)	0.0054 (10)	0.0005 (11)
C17	0.0321 (13)	0.0446 (15)	0.0516 (16)	−0.0043 (11)	0.0100 (11)	0.0035 (12)
C1	0.0298 (13)	0.0419 (15)	0.0571 (17)	−0.0047 (11)	0.0079 (12)	−0.0031 (13)
C2	0.0381 (14)	0.0407 (15)	0.0570 (17)	−0.0072 (12)	0.0082 (12)	0.0003 (13)
C18	0.0439 (15)	0.0341 (14)	0.0479 (15)	−0.0064 (11)	0.0093 (12)	0.0007 (11)
C3	0.0394 (15)	0.0409 (16)	0.0636 (18)	−0.0024 (12)	0.0011 (13)	−0.0041 (14)
C13	0.0442 (15)	0.0457 (16)	0.0442 (15)	0.0018 (12)	0.0116 (12)	−0.0026 (12)
C7	0.0525 (16)	0.0415 (15)	0.0398 (15)	0.0057 (13)	0.0180 (12)	−0.0011 (12)
C6	0.0431 (15)	0.0594 (19)	0.0591 (18)	−0.0100 (14)	0.0121 (13)	−0.0015 (14)
C4	0.0570 (18)	0.0378 (16)	0.085 (2)	−0.0051 (14)	0.0036 (17)	0.0043 (16)
C8	0.0642 (19)	0.0423 (16)	0.0556 (18)	0.0028 (14)	0.0106 (15)	−0.0011 (13)
C5	0.067 (2)	0.0508 (19)	0.074 (2)	−0.0106 (16)	0.0143 (17)	0.0181 (16)
C12	0.0607 (19)	0.0574 (19)	0.064 (2)	0.0195 (15)	0.0121 (15)	0.0002 (15)
C10	0.115 (3)	0.0354 (18)	0.100 (3)	0.008 (2)	0.038 (3)	0.0013 (18)
C9	0.087 (2)	0.0418 (18)	0.083 (2)	−0.0044 (17)	0.0135 (19)	−0.0096 (17)
C11	0.096 (3)	0.056 (2)	0.088 (3)	0.035 (2)	0.026 (2)	0.0172 (19)
O3	0.0324 (9)	0.0378 (10)	0.0620 (12)	−0.0001 (8)	0.0133 (8)	−0.0033 (8)
C20	0.0316 (13)	0.0542 (17)	0.0541 (16)	0.0054 (12)	0.0111 (12)	−0.0029 (13)
C21	0.0549 (18)	0.0573 (19)	0.0633 (19)	0.0160 (14)	0.0166 (15)	−0.0047 (15)

### Geometric parameters (Å, °)

**Table:**

Cl1—C3	1.740 (3)	C7—C12	1.385 (4)
O1—C13	1.294 (3)	C6—C5	1.379 (4)
O1—H1	1.07 (5)	C6—H6	0.9300
O2—C19	1.271 (3)	C4—C5	1.369 (5)
O2—H1	1.38 (5)	C4—H4	0.9300
C15—C19	1.400 (3)	C8—C9	1.380 (4)
C15—C16	1.414 (3)	C8—H8	0.9300
C15—C14	1.482 (3)	C5—H5	0.9300
C19—C1	1.492 (4)	C12—C11	1.378 (5)
C14—C13	1.388 (3)	C12—H12	0.9300
C14—C18	1.404 (3)	C10—C9	1.358 (5)
C16—O3	1.339 (3)	C10—C11	1.361 (5)
C16—C17	1.400 (3)	C10—H10	0.9300
C17—C18	1.366 (4)	C9—H9	0.9300
C17—H17	0.9300	C11—H11	0.9300
C1—C2	1.376 (4)	O3—C20	1.440 (3)
C1—C6	1.379 (4)	C20—C21	1.491 (4)
C2—C3	1.375 (4)	C20—H20A	0.9700
C2—H2	0.9300	C20—H20B	0.9700
C18—H18	0.9300	C21—H21A	0.9600
C3—C4	1.366 (4)	C21—H21B	0.9600
C13—C7	1.477 (4)	C21—H21C	0.9600
C7—C8	1.377 (4)		
			
C13—O1—H1	112 (2)	C5—C6—H6	119.7
C19—O2—H1	113.3 (18)	C1—C6—H6	119.7
C19—C15—C16	127.1 (2)	C3—C4—C5	118.2 (3)
C19—C15—C14	127.6 (2)	C3—C4—H4	120.9
C16—C15—C14	105.2 (2)	C5—C4—H4	120.9
O2—C19—C15	122.1 (2)	C7—C8—C9	121.0 (3)
O2—C19—C1	113.2 (2)	C7—C8—H8	119.5
C15—C19—C1	124.7 (2)	C9—C8—H8	119.5
C13—C14—C18	123.5 (2)	C4—C5—C6	120.7 (3)
C13—C14—C15	130.6 (2)	C4—C5—H5	119.6
C18—C14—C15	105.9 (2)	C6—C5—H5	119.6
O3—C16—C17	127.0 (2)	C11—C12—C7	120.5 (3)
O3—C16—C15	123.1 (2)	C11—C12—H12	119.8
C17—C16—C15	109.9 (2)	C7—C12—H12	119.8
C18—C17—C16	108.0 (2)	C9—C10—C11	119.6 (3)
C18—C17—H17	126.0	C9—C10—H10	120.2
C16—C17—H17	126.0	C11—C10—H10	120.2
C2—C1—C6	118.7 (3)	C10—C9—C8	120.4 (3)
C2—C1—C19	122.0 (2)	C10—C9—H9	119.8
C6—C1—C19	119.1 (2)	C8—C9—H9	119.8
C3—C2—C1	119.6 (3)	C10—C11—C12	120.7 (3)
C3—C2—H2	120.2	C10—C11—H11	119.6
C1—C2—H2	120.2	C12—C11—H11	119.6
C17—C18—C14	111.0 (2)	C16—O3—C20	116.93 (19)
C17—C18—H18	124.5	O3—C20—C21	107.9 (2)
C14—C18—H18	124.5	O3—C20—H20A	110.1
C4—C3—C2	122.1 (3)	C21—C20—H20A	110.1
C4—C3—Cl1	118.9 (2)	O3—C20—H20B	110.1
C2—C3—Cl1	119.0 (2)	C21—C20—H20B	110.1
O1—C13—C14	122.6 (2)	H20A—C20—H20B	108.4
O1—C13—C7	113.2 (2)	C20—C21—H21A	109.5
C14—C13—C7	124.1 (2)	C20—C21—H21B	109.5
C8—C7—C12	117.8 (3)	H21A—C21—H21B	109.5
C8—C7—C13	123.5 (2)	C20—C21—H21C	109.5
C12—C7—C13	118.6 (3)	H21A—C21—H21C	109.5
C5—C6—C1	120.7 (3)	H21B—C21—H21C	109.5
			
C16—C15—C19—O2	−174.5 (3)	C18—C14—C13—O1	−175.3 (3)
C14—C15—C19—O2	2.5 (5)	C15—C14—C13—O1	2.9 (4)
C16—C15—C19—C1	4.9 (4)	C18—C14—C13—C7	2.8 (4)
C14—C15—C19—C1	−178.2 (3)	C15—C14—C13—C7	−179.0 (3)
C19—C15—C14—C13	2.5 (5)	O1—C13—C7—C8	−137.3 (3)
C16—C15—C14—C13	179.9 (3)	C14—C13—C7—C8	44.5 (4)
C19—C15—C14—C18	−179.1 (3)	O1—C13—C7—C12	40.3 (4)
C16—C15—C14—C18	−1.6 (3)	C14—C13—C7—C12	−137.9 (3)
C19—C15—C16—O3	−0.9 (4)	C2—C1—C6—C5	−1.4 (4)
C14—C15—C16—O3	−178.4 (2)	C19—C1—C6—C5	−177.1 (3)
C19—C15—C16—C17	178.7 (3)	C2—C3—C4—C5	−0.8 (4)
C14—C15—C16—C17	1.2 (3)	Cl1—C3—C4—C5	−179.7 (2)
O3—C16—C17—C18	179.3 (2)	C12—C7—C8—C9	1.7 (4)
C15—C16—C17—C18	−0.3 (3)	C13—C7—C8—C9	179.3 (3)
O2—C19—C1—C2	−126.2 (3)	C3—C4—C5—C6	0.8 (5)
C15—C19—C1—C2	54.4 (4)	C1—C6—C5—C4	0.3 (5)
O2—C19—C1—C6	49.3 (4)	C8—C7—C12—C11	−2.2 (4)
C15—C19—C1—C6	−130.1 (3)	C13—C7—C12—C11	−180.0 (3)
C6—C1—C2—C3	1.4 (4)	C11—C10—C9—C8	−0.6 (5)
C19—C1—C2—C3	176.9 (2)	C7—C8—C9—C10	−0.3 (5)
C16—C17—C18—C14	−0.8 (3)	C9—C10—C11—C12	0.1 (6)
C13—C14—C18—C17	−179.9 (2)	C7—C12—C11—C10	1.3 (5)
C15—C14—C18—C17	1.5 (3)	C17—C16—O3—C20	6.2 (4)
C1—C2—C3—C4	−0.3 (4)	C15—C16—O3—C20	−174.3 (2)
C1—C2—C3—Cl1	178.6 (2)	C16—O3—C20—C21	170.6 (2)

### Hydrogen-bond geometry (Å, °)

**Table:**

*D*—H···*A*	*D*—H	H···*A*	*D*···*A*	*D*—H···*A*
O1—H1···O2	1.07 (5)	1.38 (5)	2.435 (3)	168 (5)
C20—H20B···O2^i^	0.97	2.51	3.246 (3)	133

Symmetry codes: (i) *x*−1, *y*, *z*.
